# Involving the microRNA Targetome in Esophageal-Cancer Development and Behavior

**DOI:** 10.3390/cancers10100381

**Published:** 2018-10-12

**Authors:** Francisca Dias, Mariana Morais, Ana Luísa Teixeira, Rui Medeiros

**Affiliations:** 1 Molecular Oncology and Viral Pathology Group, IPO-Porto Research Center (CI-IPOP), Portuguese Oncology Institute of Porto (IPO-Porto), Rua Dr António Bernardino de Almeida, 4200-072 Porto, Portugal; Francisca.Carvalho.Dias@ipoporto.min-saude.pt (F.D.); mariana.gomes.morais@ipoporto.min-saude.pt (M.M.); 2 Abel Salazar Institute for the Biomedical Sciences (ICBAS), University of Porto, Rua de Jorge Viterbo Ferreira 228, 4050-313 Porto, Portugal; 3 Research Department, LPCC—Portuguease League against Cancer (NRN Norte), Estrada Interior da Circunvalação 6657, 4200-172 Porto, Portugal; 4 Faculty of Medicine, University of Porto (FMUP), Alameda Professor Hernâni Monteiro, 4200-319 Porto, Portugal; 5 CEBIMED, Faculty of Health Sciences, Fernando Pessoa University, Praça de 9 de Abril 349, 4249-004 Porto, Portugal

**Keywords:** esophageal cancer, miR-SNPs, molecular biomarkers

## Abstract

Esophageal cancer (EC) is the eighth most common and sixth leading cause of cancer-related mortality in the world. Despite breakthroughs in EC diagnosis and treatment, patients with complete pathologic response after being submitted to chemoradiotherapy are still submitted to surgery, despite its high morbidity. Single-nucleotide polymorphisms (SNPs) in miRNA, miRNA-binding sites, and in its biogenesis pathway genes can alter miRNA expression patterns, thereby influencing cancer risk and prognosis. In this review, we systematized the information available regarding the impact of these miR-SNPs in EC development and prognosis. We found 34 miR-SNPs that were associated with EC risk. Despite the promising applicability of these miR-SNPs as disease biomarkers, they still lack validation in non-Asian populations. Moreover, there should be more pathway-based approaches to evaluate the cumulative effect of multiple unfavorable genotypes and, consequently, identify miR-SNPs signatures capable of predicting EC therapy response and prognosis.

## 1. Introduction

Esophageal cancer (EC) is the eighth most common cancer and the sixth leading cause of cancer-related mortality in the world, with an estimated 400,000 deaths in 2012 [1,2]. The vast majority of ECs occur as either esophageal squamous cell carcinoma (ESCC) in the middle or upper third of the esophagus, or as esophageal adenomarcinomas (EAC) in the distal third or esophagogastric junction [3]. EC incidence is threefold higher in men than in women, and peak incidence rates occur in Southern and Eastern Africa and in Eastern Asia [2]. The global incidence of ESCC has remained more or less the same, whereas a rapid increase in the incidence of EAC has been observed in the United States and Western Europe [3,4,5]. In fact, EAC incidence is expected to rise dramatically in many Western countries in the coming years [6,7]. EAC typically arises from a metaplastic epithelium known as Barrett’s Esophagus (BE), and established risk factors for both EAC and BE include gastroesophageal reflux disease, European ancestry, male sex, obesity, and tobacco smoking [8].

Surgical resection remains the cornerstone of curative treatment for patients with locoregional EC despite high morbidity and mortality rates due to complications, especially in older patients [9,10]. Recent randomized trials have shown that neoadjuvant chemoradiotherapy significantly improved survival in patients with resectable tumors [11,12]. As such, multimodality therapy that combines neoadjuvant chemoradiotherapy followed by surgery has become the standard of care in many institutions. However, operative morbididy and mortality associated with esophagectomy remain high, and complications arise in up to 60% of patients [13]. Additionally, 25–30% of patients experience a complete pathologic response following neoadjuvant chemoradiotherapy, but are still submitted to surgery due to the lack of accurate and reliable techniques capable of determining the complete pathological response [12,13]. This fact led some authors to question if every patient should undergo esophagectomy following chemoradiotherapy, since the subgroup that presents a complete pathological response won’t benefit from surgery and, most importantly, how patients best suited to chemoradiotherapy alone should be selected. Thus, the study and development of new biomarkers capable of predicting patients’ prognosis is imperative in order to avoid surgery and related morbidity and mortality in good responders to neoadjuvant chemoradiotherapy.

The contribution of microRNAs (miRNAs) to EC development has been extensively studied, and it has become clear that they play crucial roles in the pathogenesis, diagnosis, and prognosis of this type of cancer [14]. In fact, a study published this year by Chiam and colleagues established two miRNA-ratios (miR-4521/miR-340-5p and miR-101-3p/miR-451a) that were able to predict disease-free survival following neoadjuvant chemoradiotherapy and esophagectomy in patients with EAC [15]. MiRNAs are a class of small (~22 nt) noncoding RNAs that regulate the expression of target mRNAs at a posttranscriptional level and are implicated in various biological processes, such as embryonic development, cell differentiation, proliferation, apoptosis, and cancer development [16,17]. MiRNA biogenesis consists of a primary miRNA (pri-miRNA) undergoing cleavage in subsequently regulated steps by two enzymes to form a mature miRNA that is incorporated in an RNA-induced silencing complex (RISC), ultimately guiding it to its target mRNA to perform its regulator function [18]. Briefly, the pri-miRNA is cropped into a 55 to 80 nt stem-loop precursor miRNA (pre-miRNA) by Drosha and its cofactor DiGeorge syndrome chromosomal region 8 (DGR8) in the cell nucleus. Next, the pre-miRNA is exported to the cytoplasm by nuclear export protein (XPO5) and Dicer cleaves the pre-miRNA into a mature miRNA. The mature miRNA is then incorporated into the RISC complex, which will guide it to the complementary region of its targets. This process results either in the inhibition of mRNA translation, or in mRNA degradation, depending on the degree of complementarity between the miRNA and the 3′-UTR region of its target mRNA [16]. The crucial binding location for mRNA translational regulation resides in the mature miRNA sequence and, more accurately, within nucleotides 2–7 or 2–8 from the 5′end of the miRNA, called the seed region [18,19]. It is important to note that the average size of the human 3′-UTR is about 950 nt, while an efficient miRNA-binding site consists of 6–8 nt. As such, the 3′-UTR of a specific mRNA can include tandem target sequences for a specific miRNA as for many other miRNAs [20,21]. Since the major consequence of miRNA:mRNA pairing is the loss of protein expression, resulting from either decreased transcript levels or by translational repression, alterations in miRNA expression patterns impact on the expression of oncoproteins and tumor suppressor proteins, thereby influencing cancer risk and prognosis [20].

Given the diversity of pathways that are regulated by miRNAs, genetic polymorphisms in miRNAs, miRNA-processing machinery, and miRNA target sites are implicated in carcinogenesis. MiRNA-related single-nucleotide polymorphisms (miR-SNPs) are defined as SNPs that occur in miRNA genes, at miRNA-binding sites, and in the miRNA processing machinery. This type of SNPs can ultimately affect cancer risk, treatment efficacy and, consequently, patient prognosis by modulating both miRNAs and their targets [17]. In 2016, Nariman-Saleh-Fam and colleagues used a bioinformatics approach to provide a catalog of the most potentially disruptive EC-implicated miRNA targetome polymorphisms, along with in silico insight into the pathways affected by such variations [14]. Despite the importance of these findings, validation studies are still lacking.

MiR-SNPs seem to represent an indispensable pool of novel molecular biomarkers and have recently come into focus regarding their possible role in the development of cancer. Hence, the scope of this review is to gather and systematize the information available regarding the impact of miR-SNPs in EC development and prognosis.

## 2. Evidence Acquisition

A literature search in PubMed was conducted using the search terms “miRNA”, “polymorphisms”, “SNPs”, and “esophageal cancer”. The articles were selected by relevance of their findings, namely, the significant association of miR-SNPs and esophageal cancer. Literature analysis includes scientific papers published in the last ten years (between 2008 and 2018). Obtained scientific papers were manually curated in order to determine associations between miR-SNPs and EC. Of the 47 papers found, 17 were excluded. The exclusion criteria for the collected papers were as follows: (1) no association between miR-SNPs and EC; (2) association with a benign tumor; and (3) individual papers that were already included in meta-analysis, collected for this study. For each study, information was extracted concerning the following characteristics: the name of the miRNA, SNP rs number, SNP effect on esophageal cancer (e.g., cancer risk, prognosis, therapy response), ethnicity, type of study (e.g., case control, association study, meta-analysis) and number of cases and controls used. Since BE is an established risk for, and the only known precursor of, EAC, we included the miR-SNPs that were common to both BE and EAC in our study.

## 3. Evidence Synthesis

**Figure 1:**
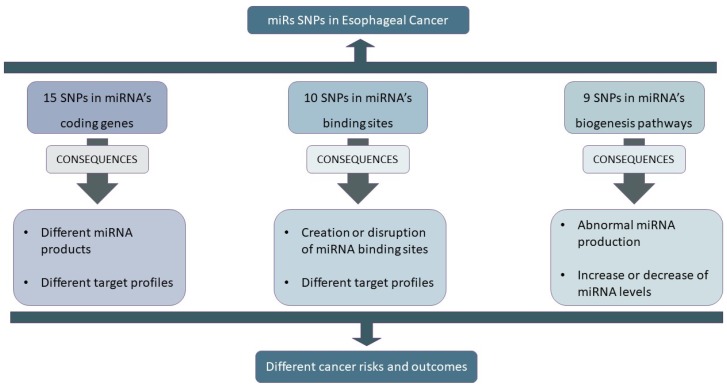
Overview of miRNA-related single-nucleotide polymorphisms (miR-SNPs) found and their impact on esophageal cancer (EC).

The pooled information is synthesized in Figure 1, where we divided the EC-relevant miR-SNPs into three categories: (1) SNPs in miRNA-coding genes, (2) SNPs in miRNA-binding sites, and (3) SNPs in biogenesis machinery.

### 3.1. SNPs in miRNA Loci

MiRNA genes are scattered among each human chromosome. With the exception of chromosome Y, they can be encoded in independent transcription units, polycistronic clusters, or within the introns of protein-coding genes [22]. MiRNA profiling has revealed that most miRNAs are significantly downregulated in cancer [20]. Calin and colleagues mapped the chromosomal location of all known miRNA genes and discovered that many are located in regions that are frequently involved in chromosomal alterations, such as deletions or amplifications, usually found in many types of cancers [23]. Additionally, Lu and colleagues found that SNPs occur less frequently, but are more constrained in miRNAs associated with diseases when compared to the other miRNAs [24]. The majority of SNPs in miRNA-coding genes usually occur in pre-miRNAs and can be responsible for changes in stem-loop structures, consequently affecting the production of mature miRNAs [25]. Gong and colleagues performed a genome-wide scan in human pre-miRNAs, miRNA flanking regions, and target sites, and their results showed that approximately 40% of pre-miRNAs contain at least one polymorphism, and 48 SNPs were found in functionally important seed regions. The authors also observed that the SNP density of pre-miRNAs is lower than that of flanking regions, and SNP density of miRNA seed regions is significantly lower than in the pre-miRNAs and flanking regions [25]. Despite being quite rare in seed sequences, SNPs in these regions can lead to the creation or disruption of putative binding sites, consequently altering the total numbers of putative targets [26].

In this review, we found 22 studies that related a total of 13 SNPs in miRNA genes associated with an EC outcome (Table 1). The most studied SNPs were miR-423 rs6505162, miR-196a-2 rs1161491,3 and miR-146a rs2910164, and they were all located in pre-miRNA regions with impact in the respective mature miRNA production. The miR-423 rs6505162 SNP is located in pre-miR-423 and maps to 17q11.2, with a nucleotide alteration from C to A [27]. Since both miR-423-3p and miR-423-5p are produced by the pre-miRNA of miR-423, it is possible for the polymorphism of pre-miR-423 to play different roles in cancer progression, and in different types of cancer [27,28,29]. Despite the lack of studies about mature miR-423 in esophageal cancer, miRNA-423-5p expression was associated with cell proliferation and invasion in gastric cancer cells [30]. The miR-146a rs2910164 G > C SNP is located in the precursor stem-loop region, opposite to the mature miRNA-146a sequence and involves a change from a G:U pair to a C:U mismatch, with an impact on mature miRNA-146a levels [31]. MiR-146a expression was reported as dramatically decreased in ESCC tissue and it was associated with a worse prognosis [32]. The miR-196a-2 rs11614913 C > T SNP is located in the stem-loop region opposite to the mature miR-196a-2, and the nucleotide change from C to T was suggested to alter the levels of the mature 3′ passenger (3p) strand of miR-196a2 and the activity of its target mRNAs [31]. The mature miR-196a was reported as upregulated in ESCC and was suggested as a potential diagnosis biomarker and therapeutic target for this neoplasia [33,34].

### 3.2. SNPs in miRNA-Binding Sites

SNPs occurring in noncoding regions can affect transcriptional regulation or post-transcriptional gene expression, thereby affecting mRNA half life and resulting in altered protein levels trough the deregulation of miRNA–mRNA binding [56]. The most widely studied SNPs in noncoding regions are the SNPs located in the 3′-UTR of mRNAs, also known as poly-miRTSs [57]. Roughly 180,000 SNPs in the human genome that are located in the 3′-UTR region were identified, along with about 2600 mature miRNA sequences that are deposited in the mirBase (v.21), which suggests that these SNPs may introduce miRNA-binding changes [20]. Additionally, by performing a genome-wide scan, Gong and colleagues found a total of 98,008 possible functional SNPs in 3′-UTRs [25]. Despite the fact that the majority of the studies about polymorphisms in miRNA targets focus on the SNPs in 3′UTRs, it is important to note that some studies revealed that miRNAs could also bind to 5′ UTRs or coding sequences of target mRNAs, suggesting that these variants could also affect miRNA regulation [58,59]. Functional SNPs in the miRNA target regions are likely to alter gene expression via affecting miRNA targeting, namely, through the creation or disruption of miRNA-binding sites. In this review, we found eight studies relating nine different SNPs in miRNA targets (8 in 3′-UTR regions and 1 in a coding sequence) with association with EC risk and outcome (Table 2).

In a study that evaluates the impact of SNP regulation of miRNA expression in colon-cancer risk, *KIAA023* rs1053667 was found to be associated with differential expression of one of its target miRNAs, miR-19b-3p, in normal colon tissue when compared to tumor tissue [65]. *Histone lysine methyltransferase (SET8)* rs16917496 results in a C to T transition that might destroy the G:C bond in the miR-502 and SET8 binding site, therefore modulating SET8 expression. The C allele is associated with a perfect complementarity with miR-502, which will lead to efficient mRNA degradation and lower SET8 protein levels. In EC, lower SET8 expression was associated with a better prognosis and survival [17]. The *Basigin (BSG*) rs11473 consists of a C to T transition that destroys the binding site of miR-483-5p at the 3′-UTR of BSG, resulting in higher mRNA levels of this gene. In a study involving EC patients, those that carried the TT genotype expressed higher levels of BSG mRNA and protein, and consequent presents higher EC risk, compared with patients with the CC genotype carriers [61]. *All-trans-retinol dehydrogenase 8 (RDH8)* encodes for a short-chain dehydrogenase/reductase enzyme involved in rhodopsin regeneration in the vision pathway and its enzymatic activity has also been linked to estrogen biosynthesis. The rs1644730 is located in the 3′-UTR of RDH8 and its predicted miR-630 binding site [8]. Given the significantly higher incidence of EAC among males versus females, a potential protective effect of estrogen has been proposed, which is in agreement with the fact that patients carrying the rs1644730 A allele presented decreased EAC risk [8]. PTPRT is a tumor suppressor that plays a crucial role in regulating tumorigenesis mechanisms [62]. Two 3′-UTR SNPs, rs2866943 C>T and rs6029959 C>A, located in the binding sites of miR-218 and miR-142-5p, respectively, were studied in EC patients, but only rs2866943 was able to disrupt the inhibitory role of miR-218 on PRPT expression and act as a protective factor in ESCC risk. Patients carrying rs2866943 CT and TT genotypes presented a small tumor size as well as the low probability of metastasis [62]. The *Erb-b2 receptor tyrosine kinase 4 (ErbB4)* gene, also known as human epidermal growth factor receptor 4 (HER4), has been reported as overexpressed in EC tissue and is also associated with TMN stage and lymph-node metastasis [66]. ErbB4 rs1595066 creates a binding site for miR-200*, a member of miR-200 tumor-suppressor miRNAs, and is associated with a lower EC risk, probably through the downregulation of ErbB4 [42]. *BRCA1* is a widely studied tumor suppressor gene and it is deregulated in several cancers. The rs799917 T>C polymorphism located in the *BRCA1* coding sequence influences miR-638-mediated regulation of BRCA1 expression [58]. BRCA1 mRNA expression analyses showed that the rs799917 C allele carriers significantly decreased BRCA1 expression in both normal and cancer esophagus tissue compared with T allele carriers, suggesting that lower BRCA1 expression may lead to a higher risk of malignant transformation of esophagus cells [58]. *MDM4* is an oncoprotein that negatively regulates p53 function. The rs4245739 A>C SNP, located in the *MDM4* 3′-UTR, creates a binding site for miR-191, resulting in decreased *MDM4* expression [63]. Rs4245739 AC and CC genotype carriers significantly decreased *MDM4* expression in normal esophagus tissue compared with AA genotype carriers, indicating consistent genotype–phenotype correlation [63]. The rs6573 SNP is a substitution from A to C, and disrupts the binding of miR-196a to *RAP1A* 3′-UTR, resulting in a higher constitutive expression of RAP1A, which is a member of the RAS oncogene family [64]. Wang and colleagues observed that RAP1A was overexpressed in ESCC tissue, and correlated with RAP1A rs6573 CC genotype and lymph-node metastasis. The authors also performed an in vitro study where they concluded that RAP1A might function as a promoter for esophageal cancer-cell migration and invasion through matrix metalloproteinase 2 [64].

### 3.3. SNPs in miRNA Processing Machinery

Although the role of miRNA’s biogenesis pathway genes in cancer development and its progression has been well established, the association between genetic variants of these pathway genes has been less studied. The occurrence of SNPs in the components of the miRNA biogenesis pathway can affect transcription, processing, transport and target gene identification, consequently affecting the overall expression of miRNAs. In this review, we found three studies relating SNPs in *XPO5*, *Gem-associated protein 3* (*GEMIN3)*, and *Gem-associated protein 4* (*GEMIN4)* genes with EC risk outcome, and all of the SNPs reported were associated with a better prognosis (Table 3).

XPO5 is a key factor in this process, as it is responsible for the nuclear export of the pre-miRNA to the cytoplasm, where it is further processed to its final miRNA conformation in order to be loaded to RNA-induced silencing complex to exert its regulatory effect [67]. It has been postulated that XPO5 miRNA regulation can be a limiting step for miRNA development since its impairment can lead to pre-miRNA trapping in the nucleolus and therefore influence cancer risk [68]. The XPO5 rs11077 consists of an A to C transition that leads to the disruption of the miR-617 binding site and the creation of a new binding site for miR-4763-5p in *XPO5* 3′-UTR, with an impact on XPO5 mRNA levels [69]. In fact, patients’ carriers of rs11077 AA genotype displayed a trend for high XPO5 expression in ESCC tissues, and these high XPO5 expression levels were also associated with high survival rates [17]. GEMIN3 and GEMIN4 are members of the GEMIN protein family, and are part of the RNA-induced silencing complex (RISC) that participates in the target RNA recognition and repression by mature miRNAs. With the exception of GEMIN4 rs910924 that is located in a 5′-UTR, all the other studied GEMIN SNPs were missense variants, meaning that they resulted in different amino acid sequences that could impact GEMIN3 and GEMIN4 protein structure and consequent miRNA regulatory function [55,70].

## 4. Discussion

Major breakthroughs in the diagnosis and treatment of EC have been achieved during the past few decades. However, the selection criteria for operative management after chemoradiotherapy are still lacking, and patients that present a complete pathologic response are still submitted to surgery. Since miRNAs are important in carcinogenesis and are capable of regulating the expression of hundreds of target mRNAs, miR-SNPs may produce more significant functional consequences and represent an ideal candidate for disease prediction.

SNPs in miRNA and miRNA-binding sites can potentially modulate miRNA–mRNA interaction and potentially create or destroy miRNA-binding sites, while those in biogenesis pathway genes can influence miRNA transcription either through altering transcription, processing, or maturation. Due to their impact in cancer development, this new class of SNPs has been widely studied, mainly due to their potential applicability as disease biomarkers. Indeed, in this review we found a total of 34 miR-SNPs that were associated, in their majority, with EC risk and were mostly studied in Asian populations where the incidence of EC is higher. However, despite promise, the impact of these miR-SNPs requires further validation, especially in non-Asian populations where these types of studies are lacking, and the incidence of EC, especially EAC, will rise dramatically in the following years. Additionally, since EC is a type of cancer that involves multiple miR-SNPs with impact on the miRNA targetome, the candidate gene approach that considers one or few genes/SNPs at a time can give us the functional impact of that genetic variant in cancer risk or survival, but fails to relate that information with the molecular pathways involved. As such, more pathway-based approaches that evaluate the cumulative effect of multiple unfavorable genotypes on the miRNA targetome are needed to identify signatures of genetic variations capable of predicting EC therapy response and prognosis. Furthermore, it is also important to take into account that sometimes changes in a gene’s mRNA levels are not reflected in its protein levels. Hence, when studying SNPs that alter miRNA:mRNA binding capacity and, consequently, mRNA regulation, they should be accompanied by the monitorization of the protein levels. This type of approaches would shed some light on the fine regulatory mechanisms by which these variations contribute to EC pathogenesis instead of only focusing in cancer risk.

## 5. Conclusions

Despite the complexity of the functional effects of SNPs that occur in noncoding regions, which is the case of miR-SNPs, more attention has been paid to this recent class of genetic polymorphisms mainly due to their potential impact in cancer. In EC, the majority of the studies focuses on the association cancer risk and overall survival, but are lacking in terms of therapy response and prognosis. Despite the potential of miR-SNPs as biomarkers, more studies are needed, especially in non-Asian populations, in order to validate their application in the clinical practice.

## Figures and Tables

**Table 1:** Single Nucleotide Polymorphisms (SNPs) within miRNA-encoding genes and their association with Esophageal Cancer (EC) risk and outcome.

Location	SNP	Population	Type of Study	Sample Size	Relevant Genotype	Outcome	References
*Pri-miR-124-1*	rs531564	Kazach	Case controls	239 cases/227 controls	CG+GG	↓ ESCC risk	Wu et al. (2018) [35]
*Pri-miR-124-1*	rs531564	Canadian	Cohort	368 cases	G allele	↑ EAC OS	Faluyi et al. (2017) [36]
*Pre-miR-423*	rs6505162	Iranian	Case control	200 cases/300 controls	AA	↓ EC risk	Nariman-Saleh-Fam et al. (2016) [14]
*Pre-miR-196a2*	rs11614913	Chinese	Case control	1400 cases/2185 controls	CC	↑ ESCC risk	Shen et al. (2016) [37]
*Pre-miR-499*	rs3746444				C allele		
*Pre-miR-4467* *Pre-miR-3117*	rs12534337 rs7526812	Mixed ethnicity	Case control	2515 EA cases 3295 BE cases 3207 controls	A allele C allele	↑ BE and ↑ EAC risk	Buas et al. (2015) [8]
*Pri-miR-124-1*	rs531564	Chinese	Meta analysis	1738 cases/1961 controls	GG	↓ ESCC risk	Li et al. (2015) [38]
*Pre-miR-219-1*	rs107822	Kazach	Case control	248 cases/300 controls	AA/A allele	↓ ESCC risk	Song et al. (2015) [39]
	rs213210				T allele		
*Pre-miR-100*	rs1834306	Kazach	Case control	248 cases/300 controls	CC/C allele	↓ ESCC risk	Zhu et al. (2015) [40]
*Pre-miR-499b*	rs10061133	Chinese	Case control	773 cases/882 controls	GG	↓ ESCC risk	Zhang et al. (2015) [41]
*Pre-miR-4293*	rs12220909				C allele		
*Pre-miR-196a-2*	rs11614913	Chinese	Case control	381 cases/426 controls	TT	↓ ESCC risk	Qu et al. (2014) [42]
*Pre-miR-499*	rs3746444	Mixed ethnicity	Meta analysis	12799 cases/14507 controls	TC+CC	↑ EC risk in the asian population	Chen et al. (2014) [43]
*Pri-miR-34b/c*	rs4938723	Mixed ethnicity	Meta analysis	7753 cases/8014 controls	CC	↓ ESCC risk in the asian population	Li et al. (2014) [44]
*Pre-miR-608*	rs4919510	Taiwan	Cohort	504 cases	GC	↑ OS and ↑ PFS in ESCC	Yang et al. (2014) [45]
*Pre-miR-146a*	rs2910164	Chinese	Cohort	378 cases	CG+GG	↑ risk of severe hematological toxicity in ESCC	Wu et al. (2014) [46]
*Pre-miR-196a2*	rs11614913				TT	↓ OS ESCC	
*Pre-miR-125a*	rs12976445				TT	↓ OS ESCC	
*Pre-miR-196a-2*	rs11614913	Chinese	Case control	597 cases/597 controls	CT+TT	↑ ESCC risk	Wang et al. (2014) [47]
*Pre-miR-146a*	rs2910164	Mixed ethnicity	Meta analysis	790 cases/814 controls	GC+GG	↑ EC risk in the asian population	Xu et al. (2014) [48]
*Pre-miR-423*	rs6505162	Chinese	Case control	629 cases/686 controls	AA	↑ ESCC risk	Yin et al. (2013) [49]
*Pre-miR-423*	rs6505162	Black ethnicity	Case control	368 cases/583 controls	C allele	↑ ESCC risk	Wang et al. (2013) [50]
*5* *′* *-UTR miR-26a-1*	rs7372209	Mixed ethnicity		197 cases/420 controls	T allele	↑ ESCC risk	
*Pre-miR-196a-2*	rs11614913	Chinese	Case control	380 cases/380 controls	CC	↓ ESCC risk in women	Wei et al. (2013) [51]
*Pre-miR-196a-2*	rs11614913	Mixed ethnicity	Meta analysis	4947 cases and 5642 controls	C allele	↑ EC risk	Wang et al. (2013) [52]
*Pre-miR-196a2*	rs11614913	Indian	Meta analysis	289 cases/309 controls	T allele	↓ OS in ESCC	Umar et al. (2013) [53]
*Pre-miR-146a*	rs2910164				C allele		
*Pre-miR-499*	rs3746444				C allele		
*Pre-miR-423*	rs6505162				A allele		
*Pre-miR-146a*	rs2910164	Mixed ethnicity	Meta analysis	772 cases/779 controls	C allele	↓ EC risk in the asian population	He et al. (2012) [54]
*Pre-miR-423*	rs6505162	Caucasian	Case control	346 cases/346 controls	AC+AA	↓ EC risk	Ye et al. (2008) [55]

EC: Esophageal Cancer; EAC: Esophageal Adenocarcinoma; ESCC: Esophageal squamous cell carcinoma; BE: Barrett Esophagus; OS: overall survival; PFS: progression-free survival; ↑: high; ↓: low.

**Table 2:** SNPs within miRNA-binding sites and their association with EC risk and outcome.

miRNA Binding Site	SNP	Population	Type of Study	Sample Size	Relevant Genotype	Outcome	References
*3* *′* *-UTR of KIAA0423*	rs1053667	Canadian	Cohort	368 cases	C allele	↑ OS in EAC	Faluyi et al. (2017) [36]
*3* *′* *-UTR of SET8*	rs16917496	Chinese	Case control	180 cases/142 controls	CC	↑ OS and ↑ Post-surgery survival in ESCC	Wang et al. (2016) [60]
*3* *′* *-UTR of BSG*	rs11473	Chinese	Case control	624 cases/636 controls	TT/T allele	↑ risk of ESCC	Li et al. (2016) [61]
*3* *′* *-UTR of RDH8*	rs1644730	Mixed ethnicity	Case control	2515 EA cases 3295 BE cases 3207 controls	A allele	↓ BE and ↓ EA risk	Buas et al. (2015) [8]
*3* *′* *-UTR of PTPRT*	rs2866943	Chinese	Case control	790 cases/749 controls	CT/TT	↓ risk of ESCC	Yao et al. (2015) [62]
	rs6029959				CC/AC	↑ risk of ESCC	
*3* *′* *-UTR of ErbB4*	rs1595066	Chinese	Case control	381 cases/426 controls	AA/A allele	↓ risk of ESCC	Qu et al. (2014) [42]
*Coding sequence of BRCA1*	rs799917	Jinan	Case control	540 cases/550 controls	CC	↑ risk of ESCC	Zhang et al. (2013) [58]
		Huaian		588 cases/600 controls			
*3* *′* *-UTR of MDM4*	rs4245739	Jinan	Case control	540 cases/550 controls	AC+CC	↓ risk of ESCC	Zhou et al. (2013) [63]
		Huaian		588 cases/600 controls			
*3* *′* *-UTR of RAP1A*	rs6573	Chinese	Case control	537 cases and 608 controls	CC	↑ risk of metastasis in ESCC	Wang et al. (2012) [64]

**Table 3:** SNPs within miRNA binding sites and their association with EC risk and outcome.

Gene	SNP	Population	Type of Study	Sample Size	Relevant Genotype	Outcome	References
*XPO5*	rs11077	Chinese	Cohort	128 cases	AA	↑ OS in ESCC	Wang et al. (2018) [17]
*GEMIN 3*	rs197412	Canadian	Cohort	368 cases	C allele	↑ OS EAC	Faluyi et al. (2017) [36]
*GEMIN 4*	rs910924	North American	Case control	346 cases/346 controls	G allele	Haplotype associated with ↓ EC risk	Ye et al. (2008) [55]
	rs2740348				C allele		
	rs7813				G allele		
	rs910925				G allele		
	rs3744741				C allele		
	rs1062923				A allele		
	rs4968104				T allele		
